# Concentration of Radiocesium in the Wild Japanese Monkey (*Macaca fuscata*) over the First 15 Months after the Fukushima Daiichi Nuclear Disaster

**DOI:** 10.1371/journal.pone.0068530

**Published:** 2013-07-03

**Authors:** Shin-ichi Hayama, Sachie Nakiri, Setsuko Nakanishi, Naomi Ishii, Taiki Uno, Takuya Kato, Fumiharu Konno, Yoshi Kawamoto, Shuichi Tsuchida, Kazuhiko Ochiai, Toshinori Omi

**Affiliations:** 1 Nippon Veterinary and Life Science University, Musashino, Tokyo, Japan; 2 Conservation and Animal Welfare Trust, Kunitachi, Tokyo, Japan; 3 Shin-Fukushima Agricultural Cooperative, Fukushima, Fukushima, Japan; 4 Primate Research Institute, Kyoto University, Inuyama, Aichi, Japan; USGS National Wildlife Health Center, United States of America

## Abstract

Following the massive earthquake that struck eastern Japan on March 11, 2011, a nuclear reactor core meltdown occurred at the Fukushima Daiichi Nuclear Power Plant, operated by Tokyo Electric Power Company, and was followed by the release of large amounts of radioactive materials. The objective of this study was to measure the concentration of radiocesium ^134^Cs and ^137^Cs in the muscle of Japanese monkeys (*Macaca fuscata*) inhabiting the forest area of Fukushima City and to determine the change in concentration over time as well as the relationship with the level of soil contamination. Cesium concentrations in the muscle of monkeys captured at locations with 100,000–300,000 Bq/m^2^ were 6,000–25,000 Bq/kg in April 2011 and decreased over 3 months to around 1,000 Bq/kg. However, the concentration increased again to 2,000–3,000 Bq/kg in some animals during and after December 2011 before returning to 1,000 Bq/kg in April 2012, after which it remained relatively constant. This pattern of change in muscle radiocesium concentration was similar to that of the change in radiocesium concentration in atmospheric fallout. Moreover, the monkeys feed on winter buds and the cambium layer of tree bark potentially containing higher concentrations of radiocesium than that in the diet during the rest of the year. The muscle radiocesium concentration in the monkeys related significantly with the level of soil contamination at the capture locations.

## Introduction

Following the massive earthquake that struck eastern Japan on March 11, 2011, a nuclear reactor core meltdown occurred at the Fukushima Daiichi Nuclear Power Plant operated by Tokyo Electric Power Company. This was followed by the release of a large amount of radioactive material from the nuclear reactor between March 12 and 15 which contaminated large areas of land. Up to 1,500 km^2^ were contaminated, and from which more than 160,000 residents were evacuated.

After the nuclear power plant disaster at Chernobyl in 1986, various studies were conducted to assess the effect of radioactive materials on both human health and the ecosystem. However, there is no consensus on the interpretation of results [1], [2]. In Fukushima Prefecture, a correlation was reported between radiation dose and the occurrence of morphological abnormalities in Lycaenidae butterflies [3] as well as decreased abundance of birds, butterflies, cicadas and spiders [4], [5], but no scientific investigation on the effect of radiation exposure in other wild animals has been conducted. We therefore started to examine the relationship between exposure to radioactive materials and the health status of wild Japanese monkey (*Macaca fuscata*) populations inhabiting the eastern part of Fukushima Prefecture, the location of the Fukushima Daiichi Nuclear Power Plant.

The Japanese monkey is endemic to Japan, is known as the world’s northernmost wild primate, and has a life span of more than 20 years [6]. The Japanese monkey usually forms a troop of 50–100 individuals of maternal lineage, and each troop has a home range of about 4–27 km^2^in snowy areas [7]–[9]. They feed mainly on plant leaves and fruits, but also eat insects and other small animals [10]. In the Japanese monkey populations of Fukushima Prefecture, females aged 6 years or more give birth in the spring, with a birth rate of about 50% in adult females [11].

**Figure 1:**
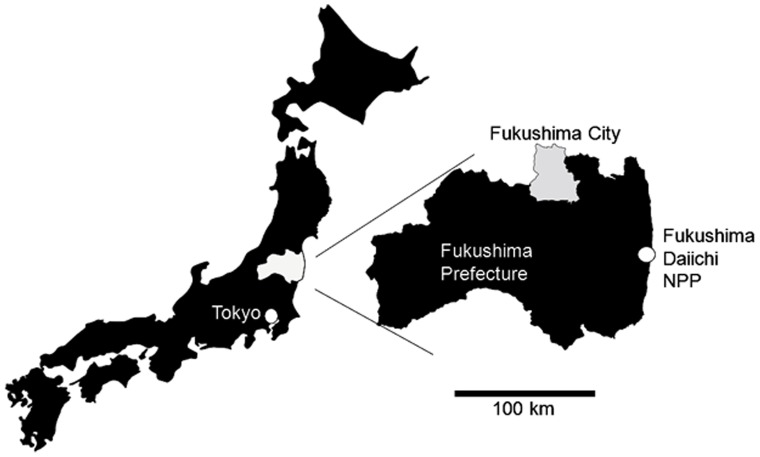
Map showing the location of Fukushima City, where the present investigation was conducted.

In the eastern part of Fukushima Prefecture, including the restricted areas, about 3,000 Japanese monkeys have been identified [12]. However, scientists are not currently permitted to enter the restricted areas for animal research. Thus, we carried out the present study with a population of Japanese monkeys inhabiting the forest area of Fukushima City, located 70 km from the Fukushima Daiichi Nuclear Power Plant (Fig. 1).

We started the research project to examine the health effect of long-term radiation exposure on wild primates, and we anticipate that our findings will provide valuable data and insight. In addition, data from non-human primates - the closest taxonomic relatives of humans - should make a notable contribution to future research on the health effects of radiation exposure in humans. For these purposes, it is necessary to characterize the change in the accumulation of radioactive materials in Japanese monkeys over time as basic data for estimating radiation exposure in these monkeys. Consequently, the objective of the present study was to determine the trend in radiocaesium activity concentration in the monkeys of the period April 2011 to June 2012. This is the first radioecological field study to monitor radiation exposure due to a nuclear disaster in a wild primate population in a fixed area over a period of 15 months.

## Methods

### Ethics Statement

Carcasses of Japanese monkeys were provided by Fukushima City. Monkeys were culled as a measure against crop damage with the permission of the governor of Fukushima Prefecture, according to the Fukushima Japanese Monkey Management Plan which was established based on the Wildlife Protection and Hunting Management Law. Monkeys were captured using box traps and killed with a gun by licensed hunters at the request of Fukushima City. The capture and killing method was in accordance with the guidelines of the management plan stated above and should not be an ethical concern. This killing method was also in accordance with guidelines published by the Primate Research Institute, Kyoto University and the International Primatological Society [13], [14]. Moreover, the Japanese monkeys inhabiting this area are not listed as endangered species on the Japanese Red List revised by the Ministry of Environment in 2012 [15].

### Animal Specimens

The carcasses of 155 Japanese monkeys collected in Fukushima City between April 11, 2011 and June 3, 2012 were assessed. The dates and locations of capture were recorded by the hunter who captured the animals. Carcasses were transported either frozen or refrigerated to Nippon Veterinary and Life Science University and subjected to autopsy as soon as possible. The body weight of each monkey was measured in grams. During necropsy, the sex and the status of tooth eruption were checked, and 500–1,000 g of muscle tissue were collected from the hind limb for measurement of radiocesium content. Collected muscle tissues were stored frozen at −30°C until used for radioactivity measurement.

**Figure 2:**
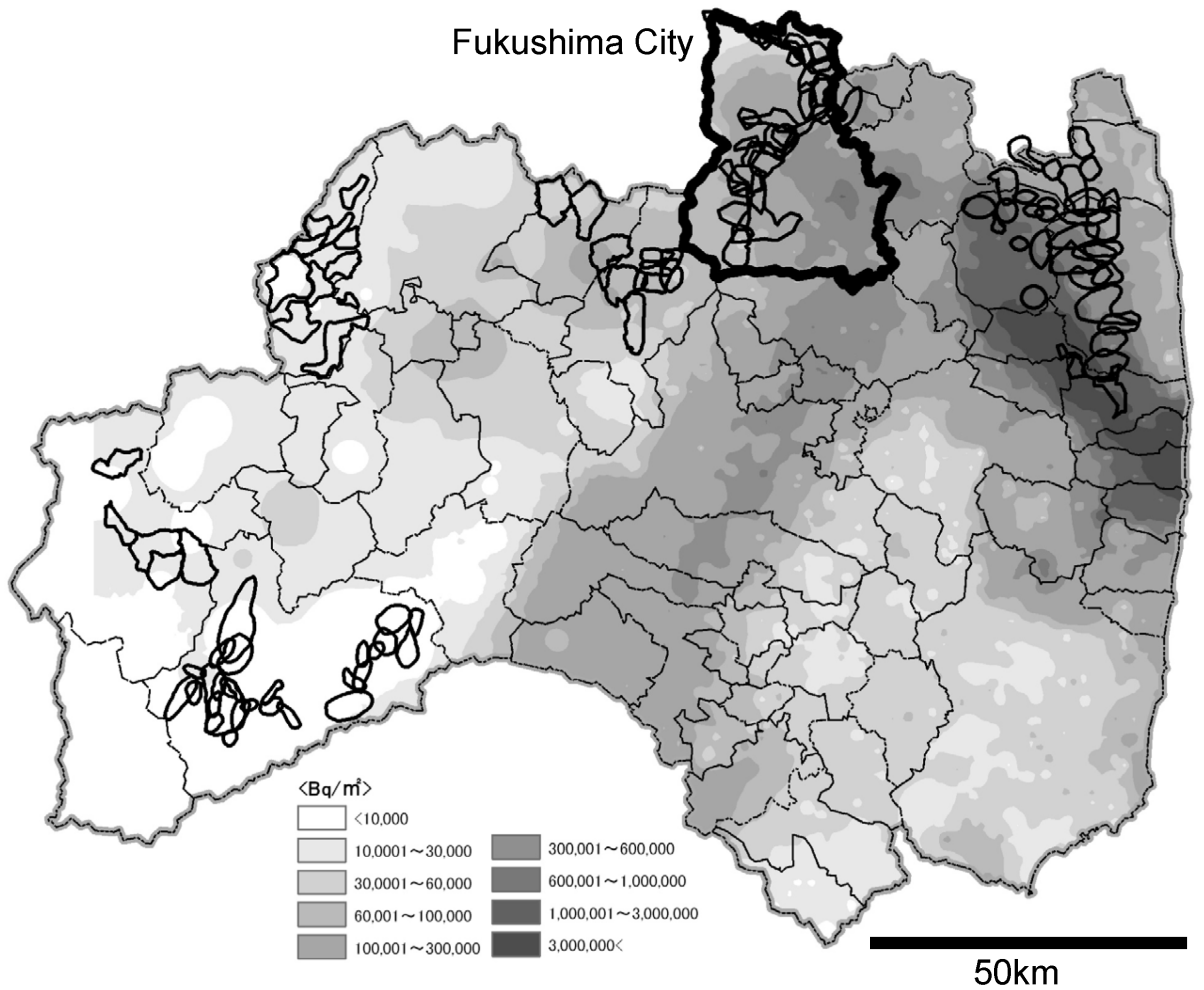
The soil contamination levels by radiocesium concentration (Bq/m^2^) and the distribution of monkey troops (irregular outlines) in Fukushima Prefecture. This map was made according to the soil contamination map created by the Ministry of Education, Culture, Sports, Science and Technology (converted to the values of July 2, 2011) [16] and the distribution map of Japanese monkeys by Fukushima Prefecture [12]. Fukushima City, the study area, is indicated by a thick outline.

The age of each animal was estimated from the status of tooth eruption, as described by Iwamoto et al. [16], to divide the animals into the following age groups: juveniles (0–3 years), subadults (4–5 years), and adults (≥6 years). The radiocesium contamination levels at each capture location were divided into 4 bands of 10,000–30,000 Bq/m^2^, 30,000–60,000 Bq/m^2^, 60,000–100,000 Bq/m^2^, and 100,000–300,000 Bq/m^2^, according to the soil contamination map in Airborne Monitoring by the Ministry of Education, Culture, Sports, Science and Technology (MEXT) [17]. We used the soil contamination map in which measured values were converted to those of July 2, 2011 (Fig. 2).

### Radioactive Measurements

Muscle samples were analyzed using a germanium semiconductor spectrometer (Canberra, GC2020-7500SL-2002CSL, Meriden, CT) and a NaI (T1) scintillation detector (Atometex, AT1320A, Minsk, Belarus). Data were corrected to background radiation dose in the measurement environment on an as-needed basis. ^134^Cs was detected using 604.70- and 795.85-keV gamma-ray energies, while ^137^Cs was detected using 661.6-keV gamma-ray energy. The radioactivity of radiocesium was adjusted to the value on the day of capture based on its physical half-life. The physical half-life of ^134^Cs and ^137^Cs is defined as 2.06 and 30.17 years, respectively. Muscle cesium concentration was calculated as the combined concentration of ^134^Cs and ^137^Cs per kg fresh weight muscle.

We measured radiocesium concentration in atmospheric fallout based on the assumption that the radiocesium contained in atmospheric fallout can contaminate or be absorbed by plants and can be subsequently orally ingested by monkeys that feed on these plants. The Fukushima Prefectural government measures the concentrations of radioactive materials in atmospheric fallout, including those in rainwater, on a daily basis at 26 monitoring sites across the prefecture. The MEXT summarizes and reports the concentration data provided by each prefecture on a monthly basis [18]. We used data from the monitoring sites in Fukushima City, where the monkeys were captured, to examine the change in radiocesium concentration in atmospheric fallout over time during the study period.

### Statistical Analysis

To compare mean muscle radiocesium concentration, one-way analysis of variance with a Bonferroni post-hoc analysis was used to determine statistical differences between multiple groups. The difference between the mean values of the two groups was analyzed using the t-test.

## Results

**Figure 3:**
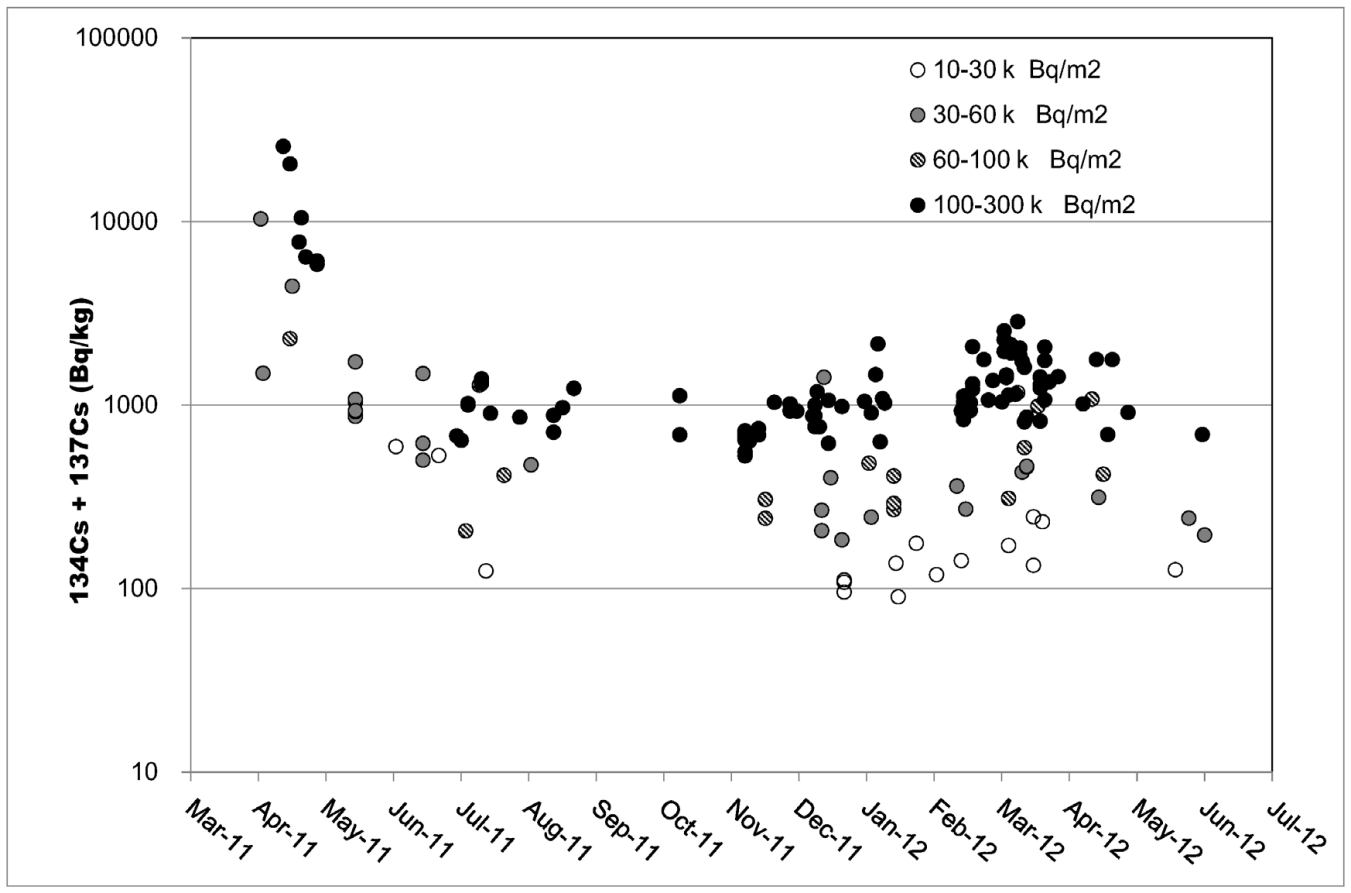
Change in muscle radiocesium concentration in Japanese monkeys over time. Individual data from animals captured in each area with soil radiocesium contamination are shown.

**Figure 4:**
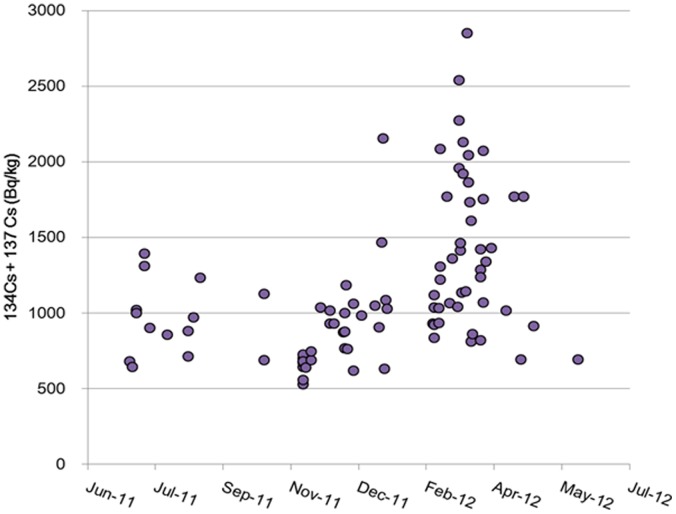
Change in muscle radiocesium concentration in Japanese monkeys captured during and after June 2011. Individual data from monkeys captured in the area with a soil radiocesium contamination of 100,000–300,000 Bq/m^2^ are shown.

Samples numbers according to contamination level, sex, and age group are summarized in Table 1.We were unable to procure carcasses of Japanese monkeys captured in March 2011, when a large amount of radioactive material was released from the Fukushima Daiichi Nuclear Power Plant, but we were provided with carcasses of monkeys captured during and after April that could be used for comparisons over time. Figure 3 shows changes in the muscle radiocesium concentration in monkeys captured in areas with soil contamination levels of 30,000–60,000 Bq/m^2^ and 100,000–300,000 Bq/m^2^. Muscle cesium concentration was relatively high in April 2011, in the range of 6,000 to 25,000 Bq/kg in the monkeys captured in areas with a soil contamination level of 100,000–300,000 Bq/m^2^, but decreased rapidly in May and June 2011 and sustained around 1,000 Bq/kg in monkeys captured during and after June, although there were substantial individual differences. However, some monkeys captured in December 2011 through April 2012 showed muscle radiocesium concentrations of 2,000–3,000 Bq/kg (Fig. 4). After April 2012, the radioactive concentration decreased to circa 1,000 Bq/kg in May and June 2012.

**Table 1:** Number of samples according to level of soil radiocesium contamination at the locations of capture, sex, and age group.

Age group	Sex	Mean bodyweight (kg)	Level of soil radiocesium contamination (Bq/m^2^)			
			10,000–30,000	30,000–60,000	60,000–100,000	100,000–300,000
Juvenile	Male	3.5	2	8	3	12
	Female	2.4	3	5	4	11
Subadult	Male	7.1	2	4	3	9
	Female	6.8	3	1	0	9
Adult	Male	11.3	2	4	2	13
	Female	10.0	4	5	4	42
Total			16	27	16	96

We compared mean muscle radiocesium concentration in monkeys captured during and after June 2011, when decreased muscle concentration was noted, in areas with 4 levels of soil contamination (Table 2). When the mean muscle radiocesium concentration was compared between the 4 areas using one-way analysis of variance with a Bonferroni post-hoc analysis, the mean muscle concentration in monkeys captured in areas with a soil contamination level of 100,000–300,000 Bq/m^2^ was significantly higher than that in monkeys captured in any of the other 3 areas (P<0.001). Moreover, in monkeys captured in areas with a soil contamination level of 100,000–300,000 Bq/m^2^, a significant difference was observed in mean muscle concentration between monkeys captured between December 2011 and April 2012, during which an increased muscle radiocesium concentration was observed (Fig. 4), and those captured in other periods (P<0.001, *t*-test).

**Table 2:** Muscle radiocesium concentration in Japanese monkeys according to level of soil radiocesium contamination at capture locations, and capture periods.

Level of soil radiocesium contamination (Bq/m^2^)	Muscle radiocesium concentration in Japanese monkeys (Bq/m^2^)						
	n	Mean	SD	Period	n	Mean	SD
10,000–30,000	16	196	150	Dec–Apr	12	147	51
				other months	4	344	254
30,000–60,000	18	475	114	Dec–Apr	12	419	329
				other months	6	586	469
60,000–100,000	15	564	371	Dec–Apr	10	601	345
				other months	5	491	450
100,000–300,000	89	1169*	489	Dec–Apr	63	1306**	501
				other months	26	836**	235

* One-way analysis of variance with a Bonferroni post-hoc analysis showed that the mean muscle radiocesium concentration in monkeys captured in areas with a soil contamination level of 100,000–300,000 Bq/m^2^ was significantly higher than that in monkeys captured in any of the other 3 areas (P<0.001).

** In monkeys captured in areas with a soil contamination level of 100,000–300,000 Bq/m^2^, a significant difference was observed in mean muscle concentration between monkeys captured between December 2011 and April 2012 and those captured during other periods (P<0.001).

We also attempted to compare muscle radiocesium concentration by sex and age group but were unable to perform statistical analysis due to substantial variations in the time of capture by sex and age group and the small number of samples for each level of soil contamination during the same period.

**Figure 5:**
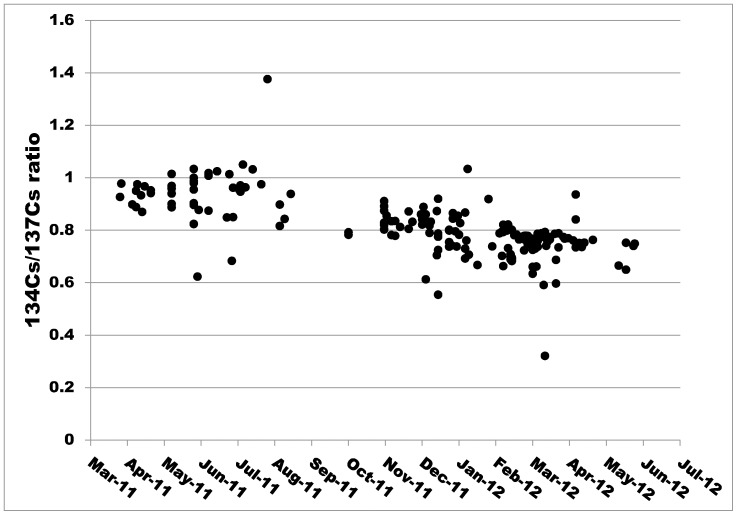
Changes in the ^134^Cs/^137^Cs concentration ratio in muscle in Japanese monkeys over time.

**Figure 6:**
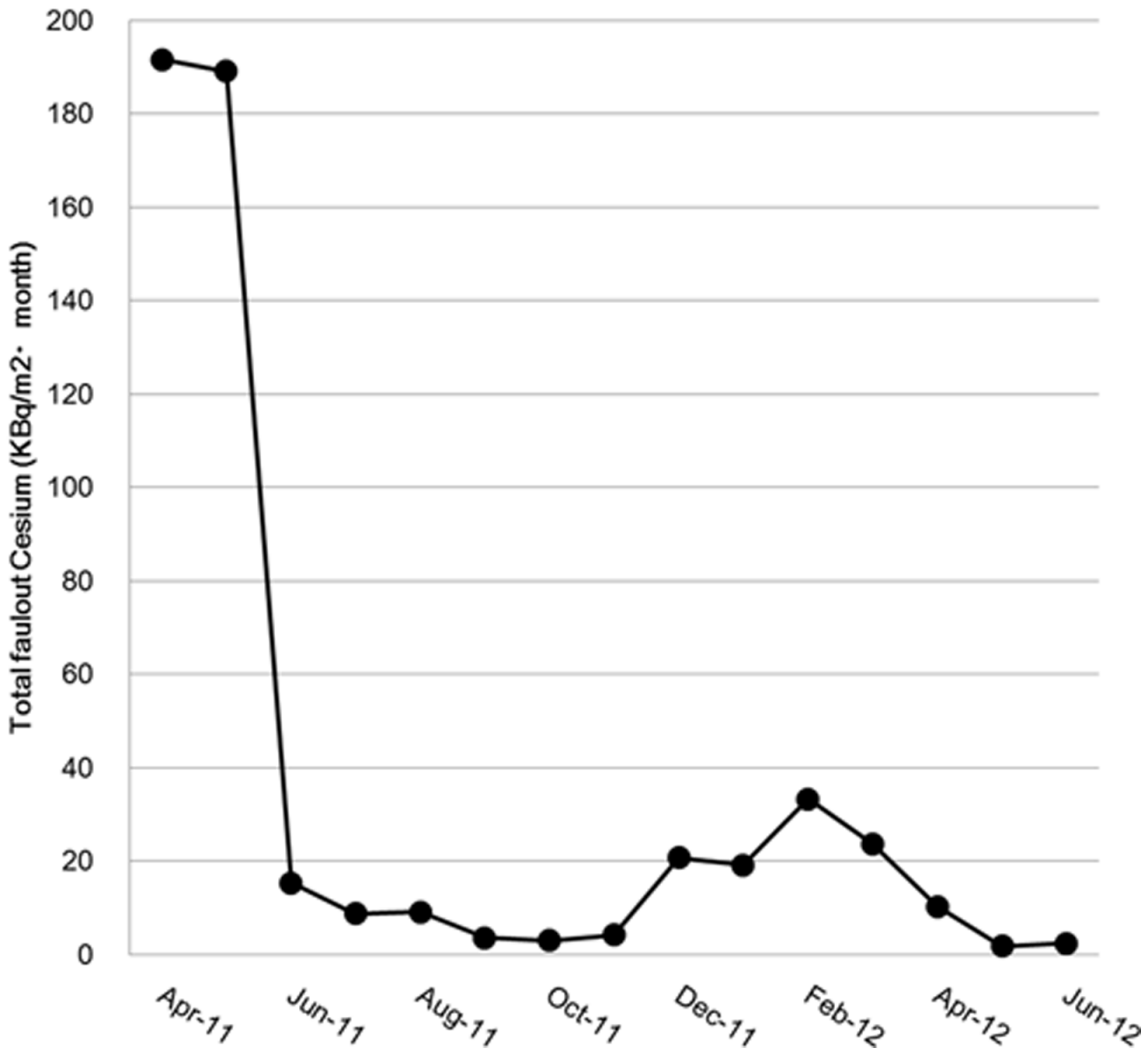
Change in monthly total concentration of radiocesium in atmospheric fallout in Fukushima City. Data published by the Ministry of Education, Culture, Sports, Science and Technology are plotted [17].

The ratio of ^134^Cs to ^137^Cs in muscle was about 0.9–1.0 in April 2011 and decreased to about 0.7 in June 2012 (Fig. 5). These values were significantly correlated with the capture date (r^2^ = 0.45, P<0.01), although a substantial inter-individual variation was found, ranging from 1.4 at maximum to 0.3 at minimum.The monthly total concentration of radiocesium in atmospheric fallout measured by Fukushima Prefecture, including that in rainwater, per unit area in Fukushima City was 6,440,000 Bq/m^2^ in March 2011. This value decreased to around 190,000 Bq/m^2^ in April and May 2011 and below 20,000 Bq/m^2^ in June 2011 and thereafter [18]. However, the level increased again to over 20,000 Bq/m^2^ between December 2011 and March 2012 before decreasing thereafter [18]. Data from April 2011 are shown in Figure 6.

## Discussion

The results of the present study revealed that the highest muscle radiocesium concentration in Japanese monkeys captured in Fukushima City was 25,000 Bq/kg in April 2011, a month after a large amount of radioactive material was released from the Fukushima Daiichi Nuclear Power Plant. In addition, the maximum concentration decreased over 3 months to about 1,000 Bq/kg, increased again to 2,000–3,000 Bq/kg in some animals in December 2011 through March 2012, and returned to 1,000 Bq/kg in April 2012, after which levels have been sustained. To date, such observational data on the muscle concentration of radioactive materials in wild mammals have not been reported for this disaster and thus provide valuable information for estimating the level of radiation exposure in wildlife.

In monkeys captured in areas with a soil contamination level of 100,000–300,000 Bq/m^2^, the mean muscle radiocesium concentration was significantly increased during winter between November 2011 and April 2012, whereas no significant seasonal change in mean muscle concentration was observed in other areas. We were unable to identify the reasons for the differences in the present study, but speculated that the small sample size of monkeys captured in less contaminated areas as well as the relatively low concentration of radiocesium accumulated in the muscle might have led to the lack of a clear seasonal change. Moreover, the fact that different numbers of monkeys were captured in different months in the area with a soil contamination level of 100,000–300,000 Bq/m^2^ also complicated the precise identification of the period with increased muscle radiocesium concentration.

The observed seasonal changes in muscle radiocesium concentration in monkeys captured in the area with a soil contamination level of 100,000–300,000 Bq/m^2^ is likely due to the change in radiocesium concentration in food items consumed by monkeys. In this study, we were unable to measure directly the level of radioactive contamination in the diet of monkeys. We therefore examined changes over time in radiocesium concentration in atmospheric fallout. This is based on our assumption that the radiocesium contained in atmospheric fallout can contaminate or be absorbed by plants and can be orally ingested by monkeys that feed on these plants. Given that more than 20,000 Bq/m^2^ of radiocesium was detected from atmospheric fallout every month between November 2011 and March 2012 (Fig. 6), it is possible that radiocesium nuclides were ingested by monkeys. The change in monthly total concentration of radiocesium in atmospheric fallout measured by Fukushima Prefecture was similar to that in muscle radiocesium concentration in the Japanese monkeys, suggesting that these monkeys continuously ingested food contaminated by atmospheric fallout from Fukushima Daiichi. The patterns of radiocesium concentration in atmospheric fallout and muscle tissue did not show a perfect correlation; there was a delay of about one month between the decrease in muscle radiocesium concentration and that in radiocesium concentration in atmospheric fallout. While the biological half-life of radioactive ^137^Cs in humans is 50–60 days [19], that in monkeys has not been determined. Beresford et al. [20] discussed the biological half-life of radiocesium in wild mammals using the formula of 13.22 M^0.2237^ taken from the second reference, based on the established relationship between biological half-life and bodyweight (M kg). When this formula is applied to the monkeys used in the present study, the biological half-life of radiocesium is estimated as 23 days, with the bodyweight of adult monkeys considered as 10 kg (Table 1), accounting for the observed time lag of about 1 month. The observed time-lag in the decrease in muscle radiocesium concentration in Japanese monkeys may be explained by the time-lag between ingesting radiocesium and its excretion from the body according to its biological half-life in Japanese monkeys.

It is also unclear why an increased radiocesium concentration in atmospheric fallout was noted between December 2011 and March 2012. During this period, neither further releases of large amounts of radioactive materials from the Fukushima Daiichi NPP nor a rise in air radiation dose were confirmed. Snowfall was one environmental change specifically observed during this period, but there has been no report of an increase in snow radiocesium concentration. Considering that the same phenomenon is observed in monitoring sites in areas with relatively low snow fall, such as Fukushima City [18]. One possible explanation is that radiocesium deposited in the soil was stirred up by the wind as dust during the winter as a result of decreased humidity. This could have resulted in a temporary increase in radiocesium concentration in atmospheric fallout.

Another possible explanation for the increased muscle radiocesium concentration during this period is that the monkeys fed on plants containing a higher concentration of radiocesium only during the winter season. In Norway, after the Chernobyl accident, some reindeer were found to have an increased muscle radiocesium concentration during the winter season [21], likely because reindeer feed on lichen containing a high concentration of cesium during winter. Similar seasonal changes in muscle radiocesium concentration have been reported in roe deer and wild boar as a consequence of changes in the diet [e.g., 22, 23]. The habitat of the Japanese monkeys examined in this study is deciduous broad-leaved forest, which receive 1–3 m of snow during winter. Therefore, Japanese monkeys in snowy areas feed mainly on winter buds and the cambium layer of tree bark during winter [24]–[26]. It has been shown that radiocesium in the soil is taken up by trees and accumulates in their winter buds and cambium layer at a relatively high concentration [27].

The relative increase in radiocesium concentration in food items that monkeys feed on in winter compared with that in other seasons may explain the observed increase in radiocesium concentration in the muscle.

The ratio of ^134^Cs to of ^137^Cs in the muscle was about 0.9–1.0 in April 2011. The fact that this ratio was reported to be 0.8–0.9 in March 2011 [28], [29] suggests that the muscle accumulation of radiocesium in monkeys occurred at almost the same ratio. However, the reason for the subsequently observed inter-individual variation in muscle radiocesium concentration could not be identified in this study.

In the present study, muscle radiocesium concentration in Japanese monkeys inhabiting the forest area in Fukushima City did not decrease over a period of at least 1 year from June 2011, but actually increased temporarily during winter. The mean muscle radiocesium concentration in monkeys captured in areas with a soil contamination level of 100,000–300,000 Bq/m^2^ was significantly higher than that in monkeys captured in areas with lower contamination levels. The level of soil contamination in the restricted area in Fukushima exceeds 3,000,000 Bq/m^2^ in some areas in which troops of wild monkey still exist (Fig.2). This contamination level is tens of times higher than that in the most severely contaminated area examined in the present study. The concentration of radiocesium in the muscle of monkeys inhabiting these areas may exceed 10,000 Bq/kg. To continuously monitor muscle radiocesium concentration for an extended period, estimate dose, and take a unique opportunity to consider health effects in wild primates, future studies should include Japanese monkeys inhabiting restricted areas, in addition to those inhabiting the forest area in Fukushima City.
